# Clinical and prognostic analysis of hepatitis B virus infection in diffuse large B-cell lymphoma

**DOI:** 10.1186/1471-2407-8-115

**Published:** 2008-04-23

**Authors:** Feng Wang, Rui-hua Xu, Hui-yan Luo, Dong-shen Zhang, Wen-qi Jiang, Hui-qiang Huang, Xiao-fei Sun, Zhong-jun Xia, Zhong-zhen Guan

**Affiliations:** 1 State Key Laboratory of Oncology in Southern China, Department of Medical Oncology, Sun Yat-Sen University Cancer Center, Guangzhou, PRoC

## Abstract

**Background:**

Hepatitis B virus (HBV) infection in diffuse large B-cell lymphoma (DLBCL) patients is a common complication in China. However, the clinical relevance of HBV infection with respect to DLBCL disease stages and patient survival remains unclear. The main objective of the current study was to analyze the clinical features and to evaluate the prognostic factors of HBV infection in DLBCL patients.

**Methods:**

In this retrospective study, DLBCL patients were divided into two groups as HBsAg-positive (n = 81) and HBsAg-negative (n = 181) patients. The HBsAg-positive patients were further divided into two subgroups based on their hepatic function during chemotherapy. Various statistical analyses were used to determine the significance of the relevant clinical parameters.

**Results:**

Compared with the HBsAg-negative group, the HBsAg-positive DLBCL group displayed a younger median onset age (46 year vs 51), more advanced stage at grade III/IV (58% vs 42%, p = 0.016), and more frequent hepatic dysfunction before (21% vs 5.5%, p < 0.001) and during (49.4% vs 16.6%, p < 0.001) chemotherapy. Female DLBCL patients exhibited a higher frequency of HBsAg positivity (p = 0.006). However, in both groups the median overall survival (OS) duration (55.8 vs 66.8 months) and response rates (91% vs 90.4%) were similar. In the HBsAg-positive DLBCL group, the poor prognostic factors were advanced stage (p < 0.001) and hepatic dysfunction during chemotherapy (p = 0.02). The OS of HBsAg-positive patients with hepatic dysfunction during chemotherapy was significantly shorter than those without liver dysfunction (p = 0.016), and the OS rates at 3 years were 48% and 72%, respectively. The use of rituximab did not increase the rates of liver dysfunction in HBsAg-positive DLBCL patients.

**Conclusion:**

Compared with HBsAg-negative patients, the HBsAg-positive DLBCL patients had earlier onset and more advanced stage. The disease stage and hepatic dysfunction during chemotherapy and were two significant prognostic factors in the HBsAg-positive DLBCL patients. This study suggests that prophylactic treatment of HBV may be of great importance in the cases of HBsAg-positive patients.

## Background

Hepatitis B virus (HBV) infection is the most common cause of chronic liver disease worldwide, and China is a highly endemic area of HBV infection with approximately 170 million carriers [1]. Previous studies have found a high prevalence of hepatitis B virus infection in non-Hodgkin's lymphoma (NHL) [2,3]. We have recently reported that HBV prevalence in B-cell NHL patients was approximately 30.2%, which was significantly higher than the HBV infection rates in T-cell NHL (19.8%) and in other cancers (14.8%) [4]. Moreover, HBsAg-positive B-cell NHL patients had an earlier disease onset than those without HBV infection. Previous studies also detected the presence of HBsAg in bone marrow cells of lymphoma [5]. Based on these findings, we hypothesized that HBV might play an important role in the development of B-cell NHL, and that the HBsAg-positive B-cell NHL patients could be considered a clinically distinct subgroup [4].

It is generally accepted that chemotherapy can reactivate prior HBV infection in patients with positive hepatitis B surface antigen (HBsAg) [6]. The reactivation rates of HBV are in the range of 20%–50% [7], and the mortality rates in these patients are 10%–40% [8]. Thus, chemotherapy-induced HBV reactivation in diffuse large B-cell lymphoma patients who are HBsAg-positive has been considered a serious complication, which is associated with long-term deterioration of hepatic function [9]. Certain therapeutic agents such as glucocorticoids and rituximab used in the chemotherapeutic regimens for diffuse large B-cell lymphoma are considered risk factors for HBV reactivation [10].

Due to the risk of HBV reactivation during chemotherapy, preemptive treatment using interferon or lamivudine (a reverse-transcriptase inhibitor of HBV DNA polymerase) is usually recommended in HBsAg-positive patients when they are undergoing chemotherapy [11-16]. However, few studies have focused on the prognosis of HBsAg-positive DLBCL patients. It is also unknown whether there is any survival benefit in patients who received anti-virus treatment. In this retrospective study, we compared the clinical features of diffuse large B-cell lymphoma patients with or without HBV infection, and evaluated the potential prognostic factors in diffuse large B-cell lymphoma patients with HBsAg-positive with stratified by various clinical features and liver function.

## Methods

### Study Samples

We analyzed diffuse large B-cell lymphoma patients who had received histological diagnosis and treatment in Sun Yat-sen University Cancer Center from January 1999 to June 2006. This retrospective study was conducted in compliance with the institutional policy to protect patient private information, and was approved by the Institution Review Board (IRB) of Sun Yat-sen University Cancer Center. Informed consents were obtained from all patients before the collection of patient information and serum samples for analyses using Enzyme-linked immunosorbent assay (ELISA) to detect hepatitis B surface antigen (HBsAg), hepatitis B surface antibody (anti-HBs), hepatitis B e antigen (HBeAg), hepatitis B e antibody (anti-HBe) and hepatitis B core antibody (anti-HBc). All patients were also tested for serum human immunodeficiency virus (HIV) antibody, HAV antibody, HCV antibody, HDV antigen, HDV antibody and HEV antibody. Patients were excluded from this study if they exhibited histological transformation from low-grade lymphoma, positive serology with HIV or HCV, previous or secondary cancer without treatment. Patients with abnormal liver tests (WHO grade >; 2) were included only if such abnormalities were related to lymphoma involvement.

### Liver function tests and definition of hepatic dysfunction

Routine liver function tests included alanine aminotransferase (ALT), aspartate aminotransferase (AST), galactosylhydroxylysyl glucosyltransferase (GGT), and bilirubin (direct and indirect forms). These assays were performed within one week before the start of each cycle of chemotherapy. Hepatic dysfunction was classified into 4 degrees according to the NCI Common Toxicity Criteria (CTC) Manual Version 2.0.

### Treatment

For early stage patients (stage I or II), four to six cycles of standard CHOP or R-CHOP regimen were given according to treatment responses and the IPI scores. After chemotherapy, patients were given regional radiation therapy (RT) to the involved body regions. For patients in advanced stages or in early stage with bulky disease, six to eight cycles of CHOP, R-CHOP or CHOP-based more aggressive regimen were given. RT was given to patients with residual disease. Patients with bulky disease in any stage were routinely given RT. Relapse or refractory patients were administrated salvage chemotherapy regimen such as EPOCH, ICE with or without rituximab. Autologous stem cell transplantation was recommended to patients when indicated. Once patients showed abnormal liver function evidenced by the tests, they were given hepatinica (diammonium glycyrrhizinate, compound glycyrrhizin, reduced glutathione, or polyene phosphatidyl choline) with or without Lamivudine. Chemotherapy continued after the liver function returned to normal.

### Response criteria

Response was evaluated according to the standard response criteria (physical examination, CT scan, and bone marrow biopsy), and imaging studies were performed every 2 cycles during chemotherapy [17]. A complete response was defined as the disappearance of all signs of disease for at least one month or an absence of change in minimal residual radiographic abnormalities for at least six months. A partial response was defined as a reduction by at least 50% in the sum of the products of the largest perpendicular diameters of all measurable lesions for at least one month. Stable disease was defined as a regression of any measurable lesion by less than 50%, or no change in the non-measurable lesions and without the growth of the existing lesions or the appearance of new lesions. Progressive disease was defined as an increase of at least 25% in the sum of the products of the largest perpendicular diameters from nadir, or a new lesion larger than 2 cm as revealed by radiography or 1 cm detected by physical examination, or involvement of the bone marrow in a patient who had a prior complete response or a clinical complete response.

### Follow-up

After treatment, routine clinical follow-up was conducted every 3 months for the first 2 years and then every 6 months for additional 3 years. Ultrasound or computed tomography scan was performed every six months during the first two years and then annually afterward during the follow-up period.

### Statistical analysis

Statistical differences in the baseline clinical parameters and treatment characteristics between the HBsAg-positive patients and HBsAg-negative patients were evaluated by either chi-square test or Fisher's exact test for categorical parameters, and Mann-Whitney U test for continuous variables. Overall survival (OS) and survival curves were calculated by Kaplan-Meier method and the differences between two groups were compared by log-rank test. OS was calculated from the date of diagnosis to the date of death or the last follow-up. The multivariate analysis of outcome in terms of OS was performed by Cox regression, which included the variables that were significant in univariate analysis. A two-tailed p value of less than 0.05 was considered statistically significant. Statistical analysis was performed with SPSS for Windows V. 13.0.

## Results

A total of 262 diffuse large B-cell lymphoma patients who received chemotherapy were included in this study. There were 81 patients in the HBsAg-positive group and 181 patients in the HBsAg-negative group. One patient in the HBsAg-positive group had HEV co-infection. All patients were HIV-negative. The baseline clinical parameters and treatment characteristics of the patients in both groups are shown in Table 1. The median follow-up time of all patients was 30 months in the HBsAg-positive group and 28 months in the HBsAg-negative group. The HBsAg-positive group was associated with advanced stage (58% HBsAg-positive vs 42% HBsAg-negative, p = 0.016), more female gender (49.4% vs 31.5%, p = 0.006), more liver involvement (9.9% vs 2.8%, p = 0.027), more spleen involvement (14.8% vs 6.2%, p = 0.024), and more hepatic dysfunction before (21% vs 5.5%, p < 0.001) or during chemotherapy (49.4% vs 16.6%, p < 0.001). The median onset age in the HBsAg-positive group was younger than the HBsAg-negative group (46 vs 51 years, p = 0.026). No significant difference was found between the two groups in their IPI scores, LDH levels, chemotherapy cycles, radiation therapy and response rates. More than 90% of the patients were given CHOP-based regimen as the first line chemotherapy in the two groups. Three HBsAg-positive patients showed grade 3 abnormal liver function and one patient with grade 4 abnormal liver function before chemotherapy, likely due to the liver involvement of lymphoma. As shown in Table 1, the occurrence of hepatic dysfunction was much higher in HBsAg-positive group than that in the negative group. Hepatic dysfunction after chemotherapy was not significantly correlated with liver function status before chemotherapy (p = 0.082). The response rates in the HBsAg-positive and -negative groups were 91% and 90.4%, respectively (p = 0.25).

**Table 1:** Comparison of HBsAg-positive patients with HBsAg-negative patients

	**HBsAg-positive patients (No = 81)(30.9%)**	**HBsAg-negative patients (No = 181)(69.1%)**	***p*-value**
**Age (years)**			0.026
Range	17–75	17–86	
Median	46	51	
**Sex**			0.006
Male	41(50.6)	124(68.5)	
Female	40(49.4)	57(31.5)	
**Stage**			0.016
I/II	34(42)	105 (58)	
III/IV	47(58)	76(42)	
**Liver involvement**			0.027
Yes	8(9.9)	5(2.8)	
No	73(90.1)	176(97.2)	
**Spleen involvement**			0.024
Yes	12(14.8)	11(6.1)	
No	69(85.2)	170(93.9)	
**LDH increase**			0.89
**> **normal	40(49.4)	91(50.3)	
normal	41(50.6)	90(49.7)	
**Extra node involvement**			0.034
≤ **1**	69(85.2)	169(93.4)	
> **1**	12(14.8)	12(6.6)	
**PS**			0.26
≤ **1**	76(93.8)	162(89.5)	
> **1**	5(6.2)	19(10.5)	
**IPI**			0.99
0,1	46(57)	103(57)	
2,3,4	35(43)	78(43)	
**Chemotherapy**			0.69
Range	1–20	1–16	
Median	6	6	
**Radiation**			0.61
Yes	26(32.1)	64(35.4)	
No	55(67.9)	117(64.6)	
**Response**			0.24
CR	43(53)	103(61.7)	
PR	31(38)	48(28.7)	
SD	5(6)	8(4.8)	
PD	3(3)	8(4.8)	
NA		14	
**Hepatic dysfunction before chemotherapy**	17(21)	10(5.5)	< 0.001
**Hepatic dysfunction during chemotherapy**	40(49.4)	30(16.6)	< 0.001

**Figure 1:**
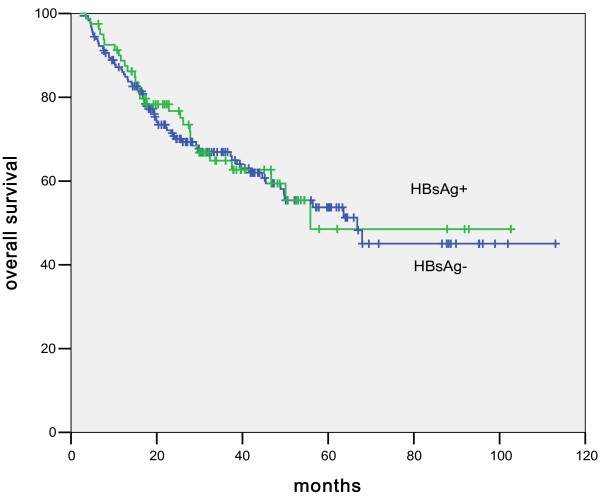
Comparison between HBsAg-positive and HBsAg-negative DLBCL patients in overall survival.

**Figure 2:**
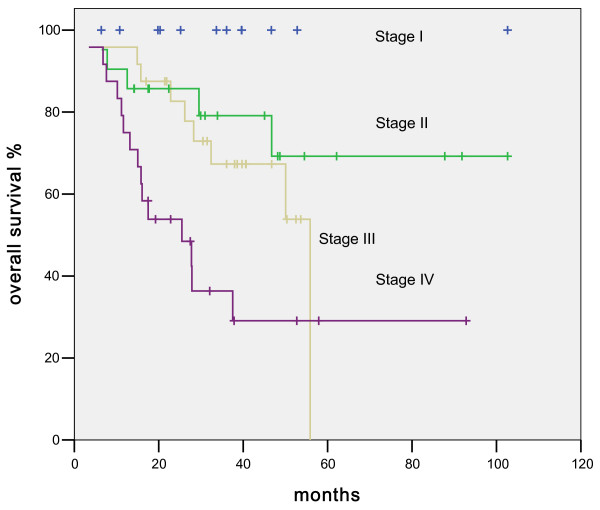
Comparison of Overall survival in HBsAg-positive DLBCL patients based on different Stages.

**Figure 3:**
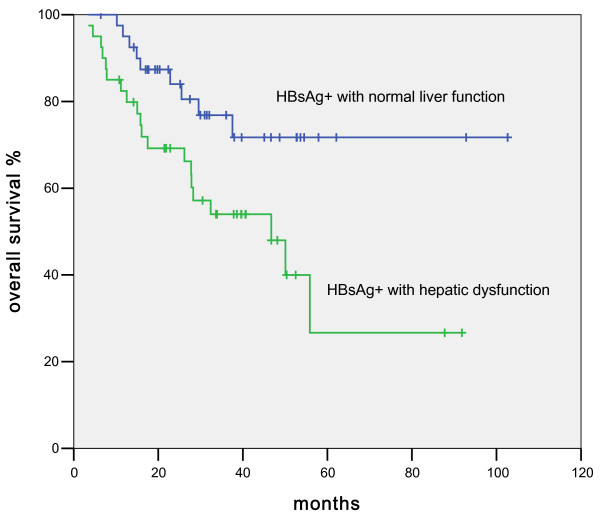
Comparison of overall survival between HBsAg-positive DLBCL patients with normal liver function and those with hepatic dysfunction during chemotherapy.

The projected median overall survival in HBsAg-positive patients was 55.8 months compared to 66.8 months in the HBsAg-negative patients (p = 0.85, Figure 1). Multiple clinical parameters were then used to analyze the prognosis of the HBsAg-positive patients and to compare the survival differences between the HBsAg-positive and -negative groups. As shown in Table 2, disease stages, radiation, and intra-chemotherapy hepatic dysfunction were significant factors that affected patient survival in HBsAg-positive patients. In the COX regression analysis, only disease stage (Figure 2) and hepatic dysfunction (Figure 3) during chemotherapy showed a significant impact on overall survival, with the p values of <; 0.001 and 0.02, respectively.

**Table 2:** Univariate and multivariate logistic regression analysis of HBsAg-positive DLBCL patients

Factor	**Median Overall survival (months)**	***p*-value in univariate analysis**	***p*-value in multivariate analysis**
**Sex**		0.96	-
Male	50		
Female	55.9		
**HBeAg**			-
+	NA	0.39	
-	55.9		
**Staging**		0.001	< 0.001
I	13		
II	21		
III	23		
IV	24		
**B symptom**		0.2	-
Yes	NA		
No	55.9		
**Age(years)**		0.21	-
**> **60	NA		
≤ 60	55.9		
**LDH**		0.17	-
**> **normal	46.7		
normal	55.9		
**Extranodal sites**		0.47	-
**> **1	37.5		
0–1	55.9		
**PS**		0.23	-
0–1	55.8		
2–4	NA		
**Radiation therapy**		0.004	0.093
Yes	NA		
No	46.7		
**Pre-CT hepatic dysfunction**		0.66	-
Yes	NA		
No	55.9		
**Intra-CT hepatic dysfunction**		0.016	0.02
Yes	46.7		
No	NA		
**Rituximab**		0.5	-
Yes	NA		
No	55.9		

Then we divided the 81 HBV-positive patients into two subgroups based on their hepatic function during chemotherapy. There were 40 patients who showed at least one time hepatic dysfunction and 41 patients who showed normal liver function during chemotherapy. The characteristics of both subgroups are shown in Table 3. The two subgroups were comparable in most clinical parameters except radiation therapy (20% v 40.5%, p = 0.037). Twenty-two patients in the hepatic dysfunction sub-group and twenty-one patients in the normal liver function sub-group received lamivudine during chemotherapy. Eight patients in the normal liver function sub-group had suffered hepatic dysfunction before chemotherapy, whose liver function turned into normal after administration of lamivudine and hepatinica. The difference in lamivudine administration between the two sub-groups was not significant (p-value 0.13).

**Table 3:** Characteristics of HBsAg-positive patients with hepatic dysfunction and without hepatic dysfunction during chemotherapy

	**Patients with hepatic dysfunction **(No = 40) (49.4%)	**Patients without hepatic dysfunction **(No = 41) (50.6%)	***p*-value**
**Sex**			0.22
Male	23(57.5)	18(43.9)	
Female	17(42.5)	23(56.1)	
**Age (years)**			0.096
≤ 60	28(70)	35(85.4)	
> 60	12(30)	6(14.6)	
**Staging**			0.088
I/II	13(32.5)	21(51.2)	
III/IV	27(67.5)	20(48.8)	
**B symptom**			0.43
Positive	15(37.5)	12(29.3)	
Negative	25(62.5)	29(70.7)	
**PS**			0.41
0,1	38(95)	37(90.2)	
2,3,4	2(5)	4(9.8)	
**LDH**			0.75
≤ Normal value	22(55)	24(58.5)	
> Normal value	18(45)	17(41.5)	
**Extranodal sites**			0.42
0–1	34(85)	32(78)	
> 1	6(15)	9(22)	
**Liver involvement**			0.63
Yes	7(17.5)	5(12.2)	
No	33(82.5)	36(87.8)	
**Spleen involvement**			0.50
Yes	12(30)	11(26.8)	
No	28(70)	30(73.2)	
**HBV status prior to CT**			0.38
HBsAg(+), HBeAg(+), Anti-HBc(+)	11(27.5)	15(36.6)	
HBsAg(+), Anti-HBe(+), Anti-HBc(+)	29(72.5)	26(63.4)	
**Liver dysfunction prior to CT**			0.38
Yes	10(25)	7(17.1)	
No	30(75)	34(82.9)	
**Radiation**			0.037
Yes	8(20)	17(41.5)	
No	32(80)	24(58.5)	
**Chemotherapy**			0.90
No. Range	2–20	1–13	
Median	6	6	
**Response to front-line chemotherapy**			0.26
CR	18(45)	25(61)	
PR	19(47.5)	12(29.3)	
SD	1(2.5)	3(7.3)	
PD	2(5)	1(2.4)	

In the HBsAg-positive DLBCL group, only one patient was not given steroid due to the abnormal liver function before chemotherapy. Four patients in the hepatic dysfunction group and five patients in the normal liver function group required dose reduction due to hepatic dysfunction or other comorbidities. Twenty-seven patients were given rituximab for at least 2 cycles, which did not increase the intra-chemotherapy hepatic dysfunction rate (Table 4). The patients received rituximab appeared to have a higher complete remission rate than those who did not (70.4% vs 44.4%). However, the statistics shows that this difference did not reach significance (p = 0.16, Table 5), probably due to the small sample size. The total numbers of chemotherapy and rituximab administration were similar between the patients with or without intra-chemotherapy liver dysfunction. Five patients were give autologous bone marrow transplantation after recurrence. Three of them exhibited hepatic dysfunction and two of them showed sustained normal liver function during chemotherapy.

**Table 4:** Hepatic dysfunction occurrence rates based ons Rituximab administration

**Use of rituximab**	**Patients with hepatic dysfunction **(N = 40) (49.4%)	**Patients without hepatic dysfunction **(N = 41) (50.6%)	***p*-value**
**Yes**	13 (32.5)	14(34.1)	0.88
**No**	27(67.5)	27(65.9)	

**Table 5:** Comparison of Response in HBsAg-positive patients treated with or without rituximab

	**Use of rituximab **(N = 27) (33.3%)	**No rituximab **(N = 54) (66.7%)	***p*-value**
**Complete response**	19 (70.4)	24(44.4)	0.16
**Partial response**	6 (22.2)	25(46.3)	
**Stable disease**	1(3.7)	3(5.6)	
**Progressive disease**	1(3.7)	2(3.7)	

The response rates to chemotherapy in the hepatic dysfunction subgroup and normal liver function subgroup were similar (92.5% vs 90.3%). Among the patients who had hepatic dysfunction during chemotherapy, the percentage of hepatic dysfunction with grade 1 to grade 4 was 30%, 30%, 10% and 30% respectively. Thirteen of them had delayed chemotherapy (1–4 weeks, median 2 weeks) because of abnormal liver function. In the HBsAg-positive patients who experienced hepatic dysfunction, ten patients stopped receiving chemotherapy because of severe abnormal hepatic function with a median 5 cycles of chemotherapy. In these 10 patients, 6 achieved CR and 4 achieved PR at the abeyance. During follow up, 4 out of 6 CR patients eventually had disease recurrence. Fourteen patients in the hepatic dysfunction subgroup and twelve patients in the normal liver function subgroup were given second-line chemotherapy after disease progression or recurrence.

**Figure 4:**
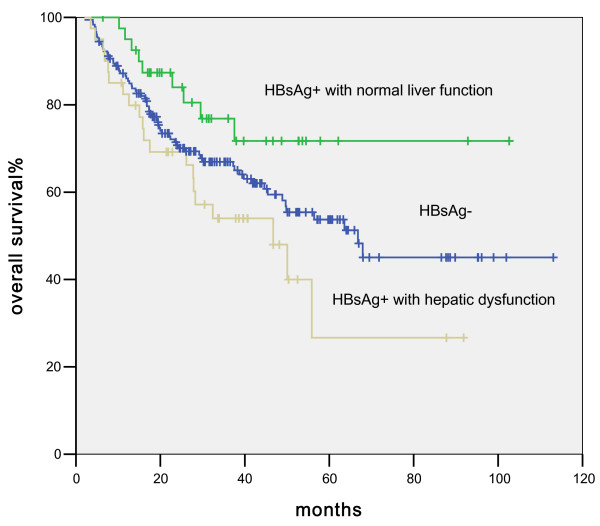
Comparison of survival differences among HBsAg-negative DLBCL patients, HBsAg-positive DLBCL patients with normal liver function, and HBsAg-positive DLBCL patients with hepatic dysfunction during chemotherapy.

The HBsAg-positive patients with hepatic dysfunction during chemotherapy had a shorter median OS (46.7 months, 95% CI of 23.5-70 months, p = 0.016, Figure 4), while the median OS of patients with normal liver function have not been reached. The overall survival rates at 3 years were 48% in hepatic dysfunction patients and 72% in normal hepatic function group. The impact of liver dysfunction on OS remained significant (p = 0.02) in the multivariate analysis (RR = 2. 6; 95% CI, 1.16-5.8). Another factor that significantly affected OS was advanced stage (RR = 2.5; 95% CI, 1.53-4.1). Other factors such as age, gender, radiation therapy, and LDH were not statistically significant.

As shown in Figure 4, the overall survival of HBsAg-positive patients with normal liver function appeared higher than that of HBsAg-negative patients. Nevertheless, this difference did not reach statistical significance (p = 0.09), likely due to the relatively small sample size. Likewise the survival difference between HBsAg-positive patients with liver dysfunction and HBsAg-negative patients was not significant. (p = 0.6)

## Discussion

In this study, the proportion of HBsAg-positive cases in diffuse large B-cell lymphoma patients was 30.1%, which was consistent with our previous observation [4]. The prevalence of HBV infection among DLBCL patients in our study was comparable with the results reported by two other groups [18,19]. The incidence of hepatic dysfunction in HBsAg-positive diffuse large B lymphoma patients was nearly 49.4%, which was similar to another report in Chinese patients [20]. HBsAg-positive DLBCL patients also had an earlier median onset age. Moreover, we found that those patients displayed higher proportion in advanced disease stages, liver or spleen involvement, and hepatic dysfunction before and during chemotherapy. A higher proportion of these patients were females. However, the overall survival in patients with and without HBV infection showed no difference. In the report by Soon-Thye Lim *et al*, the characteristics of HBsAg-positive patients with lymphoma were also similar to those who were HBV-negative in terms of age, ECOG, extra-nodal involvement, LDH level, stage, complete remission rate, and overall survival [21].

We also compared the characteristics between patients with hepatic dysfunction during chemotherapy and those without hepatic dysfunction in HBsAg-positive DLBCL patients. Although only radiation was significantly different, there was a trend that patients with older age or more advanced stage were more likely to develop hepatic dysfunction during chemotherapy. No previous study had focused on the long-term survival in HBsAg-positive NHL patients. In the current study, we demonstrated that the diffuse large B-cell lymphoma patients with hepatic dysfunction during chemotherapy had a shorter overall survival compared with those with normal liver function during chemotherapy. Our COX analysis showed that earlier stage and continuous normal liver function during chemotherapy were factors associated with longer overall survival. As shown in Figure 3, in the HBsAg-positive DLBCL group, the OS were different between patients with and without intra-chemotherapy hepatic dysfunction. However, the p value of 0.09 was not statistically significant, likely due to small number of patients in this study. The mechanism of shorter OS in patients suffering hepatic dysfunction during chemotherapy remains unknown. In some of the patients with hepatic dysfunction, the scheduled chemotherapy was incompliant due to various reasons, which might contribute to suboptimal treatment. When compared with the hepatic dysfunction patients, the normal liver function patients had higher complete remission rate (61% vs 45%).

Previous studies suggest that deterioration in liver reserve developed more frequently in patients with HBV reactivation [12,22]. In our study, among the forty patients who suffered from hepatic dysfunction during chemotherapy, seventy-five percent of them had normal liver function before treatment. This result suggested that abnormal liver function during chemotherapy may be related to chemotherapy instead of deteriorated liver function before chemotherapy. The hepatic dysfunction during chemotherapy may be a reflection of the reactivation of HBV [23]. Nevertheless, the impact of the HBV reactivation on DLBCL patients is still not fully understood [24-27].

It is commonly recognized that HBV reactivation was more likely to develop in patients with NHL than with other malignancies. Hepatitis provoked by reactivation of HBV is a well recognized complication in lymphoma patients with chronic HBV infection undergoing cytotoxic or immunosuppressive therapy.

Some reports suggest that rituximab treatment may deplete CD20+ B-cells and lead to impaired humoral immune responsiveness [28]. Other studies revealed that patients with HBV infection developed severe reactivation or fulminating hepatitis after administration of rituximab [29,30]. Because B lymphocytes can act as antigen-presenting cells during the immune attack against HBsAg-positive hepatocytes, depletion of B-cells by rituximab might play a role in HBV evading immune recognition and clearance, and thus might contribute to the viral persistence. However, in our study, administration of rituximab did not increase the incidence of intra-chemotherapy hepatic dysfunction and the addition of rituximab to chemotherapy regimens did not affect patient tolerance to chemotherapy. It seems that R-CHOP administration is safe for the HBsAg-positive DLBCL patients in our study. Masahiro Kami also reported that most patients with HBV infection tolerated rituximab plus chemotherapy without developing major adverse events [31]. Since we did not test liver function regularly after the chemotherapy was completed, it is unclear if in these patients there were undetected delayed HBV reactivation, which are known to occur in other studies [32,33].

An Italian group has shown that hepatitis C virus-positive diffuse large B-cell lymphoma patients with no hepatic dysfunction at diagnosis displayed a higher 5-year OS compared to patients with hepatitis or cirrhosis, but these differences did not reach statistical significance [34]. We found no survival difference between patients with and without hepatic dysfunction before chemotherapy. Likewise, we did not observe any difference in incidence of reactivation of HBV between hepatitis B surface antigen-positive and hepatitis B surface antigen-negative patients as suggested by another study [20].

Lamivudine has been identified as a useful drug in prevention of HBV reactivation and hepatitis [11,14,16]. In our retrospective analysis, we did not observe that the prophylactic use of lamivudine could reduce the rate of hepatic dysfunction, possibly due to suboptimal use of lamivudine at the time when prophylactic use of this drug had not been standardized. It was reported that irregular use or cessation of lamivudine treatment during chemotherapy might activate the hepatitis B virus [33]. Nevertheless, the lamivudine administration did contribute to the reduction of abnormal liver function markers after hepatic dysfunction occurred. Prospective studies are required to determine the role of lamivudine in the prevention of hepatic dysfunction.

## Conclusion

The current study showed a younger onset age, higher portion of female, liver or spleen involvement, more advanced disease in the HBsAg-positive DLBCL patient group. There was a higher incidence of hepatic dysfunction before or after chemotherapy in HBsAg-positive DLBCL. Moreover, a significant difference in overall survival was observed between patients with continuous normal liver function and those with hepatic dysfunction during chemotherapy. We propose that giving prophylactic treatments to prevent hepatic dysfunction during chemotherapy may prolong the overall survival of HBsAg-positive DLBCL patients. The HBsAg-positive B-cell NHL patients may be considered as a special patient entity who may benefit from such treatment protocol.

## Competing interests

The authors declare that they have no competing interests.

## Authors' contributions

FW and RHX were responsible for data collection and analysis, interpretation of the results, and writing the manuscript. HYL, DSZ were responsible for conducting the data analysis in cooperation with RHX, WQJ, HQH, XFS, and ZJX. ZZG was responsible for experimental design. All authors have read and approved the final manuscript.

## Pre-publication history

The pre-publication history for this paper can be accessed here:


